# Effect of Kinetic Control Training and McKenzie’s Approach on Pain and Mobility in Cervical Derangement Syndrome: A Comparative Study

**DOI:** 10.7759/cureus.89541

**Published:** 2025-08-07

**Authors:** Shamika S Baraskar, Sandeep Shinde

**Affiliations:** 1 Musculoskeletal Sciences, Krishna College of Physiotherapy, Krishna Vishwa Vidyapeeth, Krishna Institute of Medical Sciences Deemed To Be University (KIMSDU), Karad, IND

**Keywords:** cervical vertebrae, chronic pain, intervertebral disc displacement, neck pain, rehabilitation

## Abstract

Background: Cervical derangement syndrome (CDS), a form of mechanical neck pain, arises from poor posture, repetitive stress, and segmental dysfunction, resulting in discomfort, restricted cervical mobility, and reduced functional capacity. The study focuses on changes associated with CDS, particularly range of motion (ROM), pain, and functional disability. The study aims to find the effect of kinetic control training (KCT) and the McKenzie approach on CDS. The McKenzie approach emphasizes symptom centralization through repeated directional movements, while KCT focuses on restoring movement efficiency via motor control retraining. This study compares their effectiveness in managing CDS.

Objectives: The study aimed to determine the effect of KCT and the McKenzie approach on pain, functional disability, and ROM in individuals suffering from CDS.

Methods: A comparative study was conducted involving 112 participants diagnosed with CDS. Participants were randomly allocated into two groups using the envelope method. Group A (McKenzie) and Group B (KCT), with 56 participants in each group initially. Following the exclusion of six participants, the final sample consisted of 53 participants in Group A and 53 in Group B. Both groups received baseline conventional therapy followed by six weeks of their respective interventions. Pre- and post-intervention outcome measures included the Visual Analog Scale (VAS), Neck Disability Index (NDI), and cervical ROM. Statistical analysis was conducted using paired and unpaired t-tests via IBM SPSS Statistics, version 26.0 (IBM Corp., Armonk, NY).

Results: The findings demonstrated statistically significant enhancements in pain intensity, functional capacity, and cervical ROM in both groups. But Group B (KCT) demonstrated superior outcomes (p < 0.0001). In Group B (activity), VAS, NDI, and ROM improved significantly in all directions compared to Group A (McKenzie).

Conclusion: The study concluded that a six-week intervention of KCT is more effective than the McKenzie approach in reducing pain and enhancing functional capacity and cervical mobility in individuals with CDS.

## Introduction

Cervical disorders are common musculoskeletal conditions that can present with a wide range of symptoms, including neck pain, radiculopathy, tinnitus, headache, nausea, gastrointestinal discomfort, hypomnesia, palpitations, and visual dysfunction [1,2]. These symptoms can significantly impair quality of life and work capacity, contributing to growing socioeconomic and healthcare burdens. Cervical derangement syndrome (CDS), a subtype of mechanical neck pain, arises from altered joint surface alignment and may be classified as reducible or irreducible based on symptom response to directional movements [3]. It is characterized by the displacement of an intra-articular structure, such as an intervertebral disc or joint surface, from its normal anatomical position [4]. This mechanical disruption can cause pain, stiffness, and reduced range of motion. The condition may persist if individuals continue to adopt postures or movement patterns that worsen the misalignment [5]. In cervical disc derangement, the disc material shifts out of place, often from prolonged neck bending, causing pain, restricted range of motion (ROM), and nerve involvement [4]. A reduction in cervical lordosis, often due to poor posture during prolonged screen use, further predisposes individuals to posterior disc migration and symptomatic aggravation [6,7].

The symptoms of CDS may include reduced cervical lordosis, neck movement, and pain radiating to the upper back or extremities [8]. The McKenzie Mechanical Diagnosis and Therapy (MDT) model categorizes derangements by symptom distribution, response to movement, and postural deformity [9]. Additionally, thoracic spine dysfunction often coexists with CDS, where thoracic manipulation has been shown to modulate neural pathways and enhance grip strength [10]. Postural deviations, including forward head posture and rounded shoulders, further destabilize cervical biomechanics and may lead to secondary thoracic and lumbar dysfunctions. Sustained poor posture can also contribute to postural syndrome, characterized by mechanical loading-induced discomfort.

The McKenzie approach focuses on the symptom patterns observed during repeated spinal movements to classify mechanical disorders into derangement, dysfunction, or postural syndromes. Centralization is defined as the movement of pain toward the midline during specific directional loading. This phenomenon is thought to occur because repeated movements in the direction that reduces pain help to correct the position of a displaced structure inside the joint, such as an intervertebral disc [11]. Restoring the normal alignment in this way reduces pressure on pain-sensitive tissues and nearby nerves. The presence of centralization is generally considered a positive sign, as it indicates that the mechanical treatment is effective and that the patient is more likely to recover [12]. Conversely, peripheralization, the distal migration of symptoms, may signal worsening mechanical or neural involvement [13].

Kinetic control (KC), developed in the 1990s, is a movement-based clinical framework designed to retrain altered movement mechanics through low-load, high-repetition exercises that focus on deep segmental stabilizers [14]. KC training (KCT) is a therapeutic approach aimed at identifying and addressing specific movement patterns that demonstrate inadequate joint control and contribute to mechanical stress or musculoskeletal discomfort. By enhancing the functional contribution of stabilizing muscles, KCT facilitates improved joint guidance throughout movement. The intervention employs targeted exercise strategies to restore precise muscle coordination, optimize joint alignment during functional tasks, minimize undue mechanical load on soft tissues, and reduce the likelihood of recurrent pain or dysfunction in the affected region. It emphasizes the functional integration of joint-specific movement strategies in daily tasks to enhance joint stability, improve posture, and support effective pain management [15]. The McKenzie approach utilizes repeated movements in a specific direction to reduce derangement and achieve symptom centralization, while KCT focuses on addressing altered movement kinematics by restoring joint stability, enhancing movement coordination, and optimizing the function of local stabilizing muscles. Although both approaches are used in clinical practice to address mechanical neck disorders, there is limited evidence directly comparing their effectiveness in individuals with CDS. This study aims to assess and compare the outcomes of KCT and the McKenzie approach on pain, cervical mobility, and functional performance in individuals with CDS. 

## Materials and methods

Study participants and sampling

This was a comparative study that was commenced after approval from the Institutional Ethical Committee of Krishna Institute of Medical Sciences, Karad, India (approval number: 025/2023-2024), with 112 participants using the computerized IBM SPSS Statistics software, version 26.0 (IBM Corp., Armonk, NY) for data analysis. Using the following formula, the sample size was estimated to be 112, where prevalence (p) was 7%, q (100-p) was 93%, and allowable error (L) was 5.

$n = \frac{4 \cdot p \cdot q}{L^{2}}$

**Figure 1:**
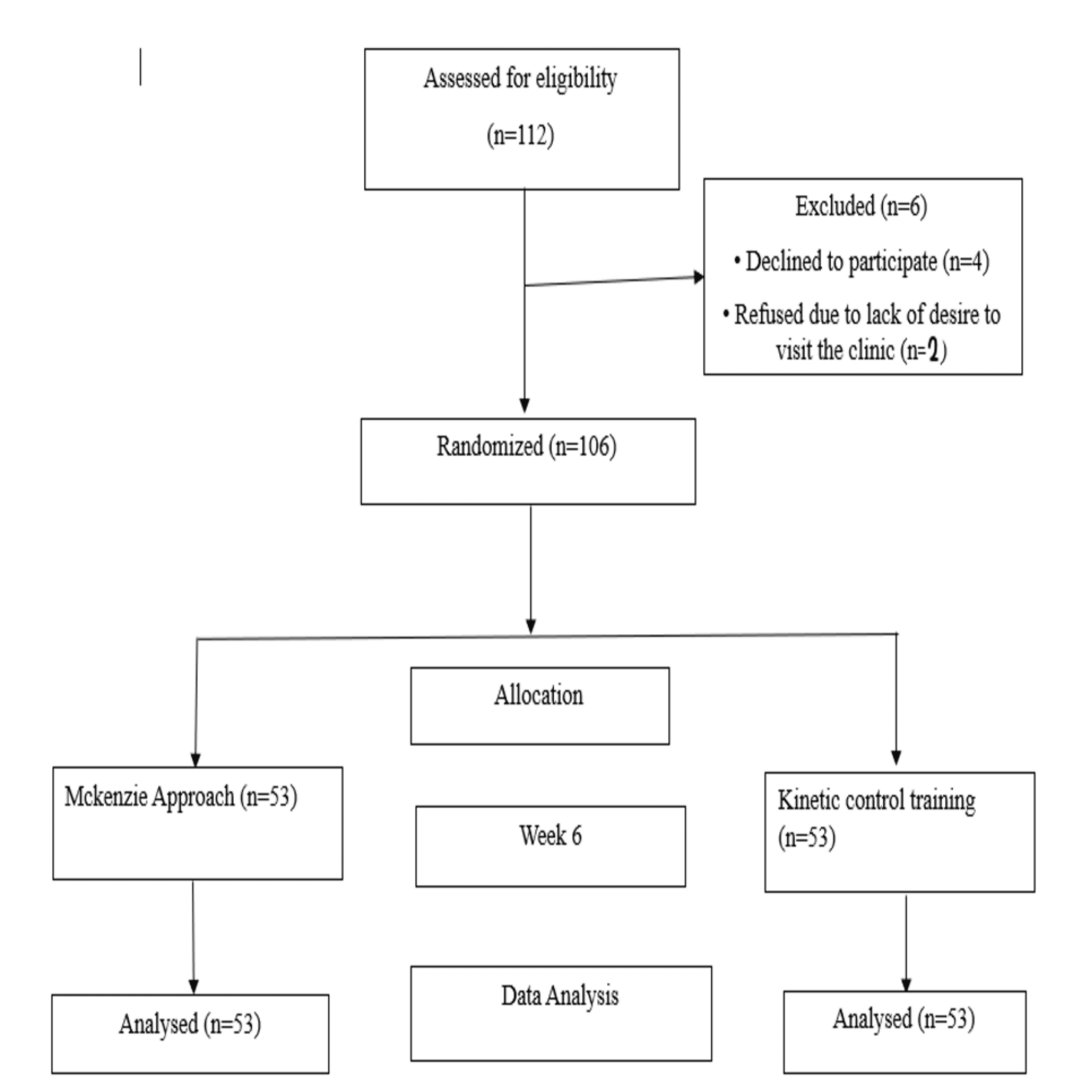
Study flowchart

The intervention was implemented over a period of six weeks. It was a single-blinded study where participants were blinded. The study population comprised both male and female individuals, aged 30 to 50 years, who had a confirmed clinical diagnosis of cervical derangement syndrome and associated neck pain. Eligibility criteria additionally required the presence of unilateral radiating pain extending into the upper limb. Diagnosis was established using the MDT classification system. Furthermore, only participants who had been experiencing neck pain for a duration exceeding 90 days and who reported a minimum pain intensity score of three or higher on the Visual Analog Scale (VAS) were included. Patients were excluded from the study if they presented with a prior history of cervical or spinal surgery, a recent traumatic event or fracture, congenital abnormalities of the cervical spine, or any diagnosed psychiatric disorder or substance abuse. The participants were randomly assigned to two groups. Participants were randomly allocated to two groups using a simple randomization technique. Allocation was done using the envelope method, where two envelopes labelled as Group A and Group B were prepared. Before the start of the intervention, each participant was instructed to pick one envelope, which determined their group assignment. This method ensured an impartial and unbiased distribution of participants across both groups. Group A, which underwent MDT exercises, and Group B, which participated in KCT (Figure 1). Participants were allocated according to inclusion and exclusion criteria. Before starting the study, all participants received a detailed explanation of the research procedures and objectives. They were then asked to provide written informed consent, confirming their voluntary participation and understanding of the study protocol. All sessions were supervised by a trained physiotherapist.

Outcome measures

VAS

It is a measurement instrument designed to assess a characteristic believed to vary along a spectrum of values and is not easily quantifiable through direct methods. The VAS is a unidimensional measure of pain intensity that has been extensively employed in a wide range of adult populations [16,17]. Validity of VAS measured by intraclass correlation coefficient was 0.97 (95% CI = 0.96 to 0.98).

Neck Disability Index (NDI)

NDI is a condition-specific functional status questionnaire with 10 items, including pain, personal care, lifting, reading, headaches, concentration, work, driving, sleeping, and recreation. A higher score indicates more patient-rated disability [18]. Validity of NDI measured by the intra-class correlation coefficient was 0.88 (0.63 to 0.95).

ROM Analysis

Cervical ROM is commonly utilized as an outcome measure in clinical trials involving individuals with cervical derangement syndrome. ROM assessment is considered a reliable and valid method for evaluating spinal mobility, including lumbar range of motion, and serves as a practical and accessible outcome measure in both clinical research and physiotherapy practice [19].

Treatment protocol

All the participants, before filling out the consent form, were briefed on the purpose and methodology of the study. In this research, 112 participants were selected, whose MDT assessment was taken to confirm CDS. They had been put on the treatment for six weeks with four sessions a week. After finding the suitability of the subjects as per the inclusion and exclusion criteria. Informed consent was obtained from the participants who were willing to participate and were recruited for the study. The participants were categorized into two groups, with 56 participants in each group, through a sealed envelope to categorize the individuals in the two groups by a randomized allocation sequence. All the subjects were assessed for neck pain using the VAS, the NDI, and ROM. The subjects were divided into two groups based on inclusion and exclusion criteria. Both groups received a baseline conventional treatment consisting of a hot moist pack applied for 15 minutes, interferential therapy (IFT) administered with a frequency of 4,000 Hz and a beat frequency of 100 Hz for 15 minutes, followed by supervised neck isometric exercises targeting flexors, extensors, and lateral flexors, each held for five to 10 seconds and repeated for 10 repetitions per direction. Participants in Group A underwent the McKenzie approach exercises [20, 21], while the participants in Group B underwent KCT [22].

Group A: McKenzie Approach

**Figure 2:**
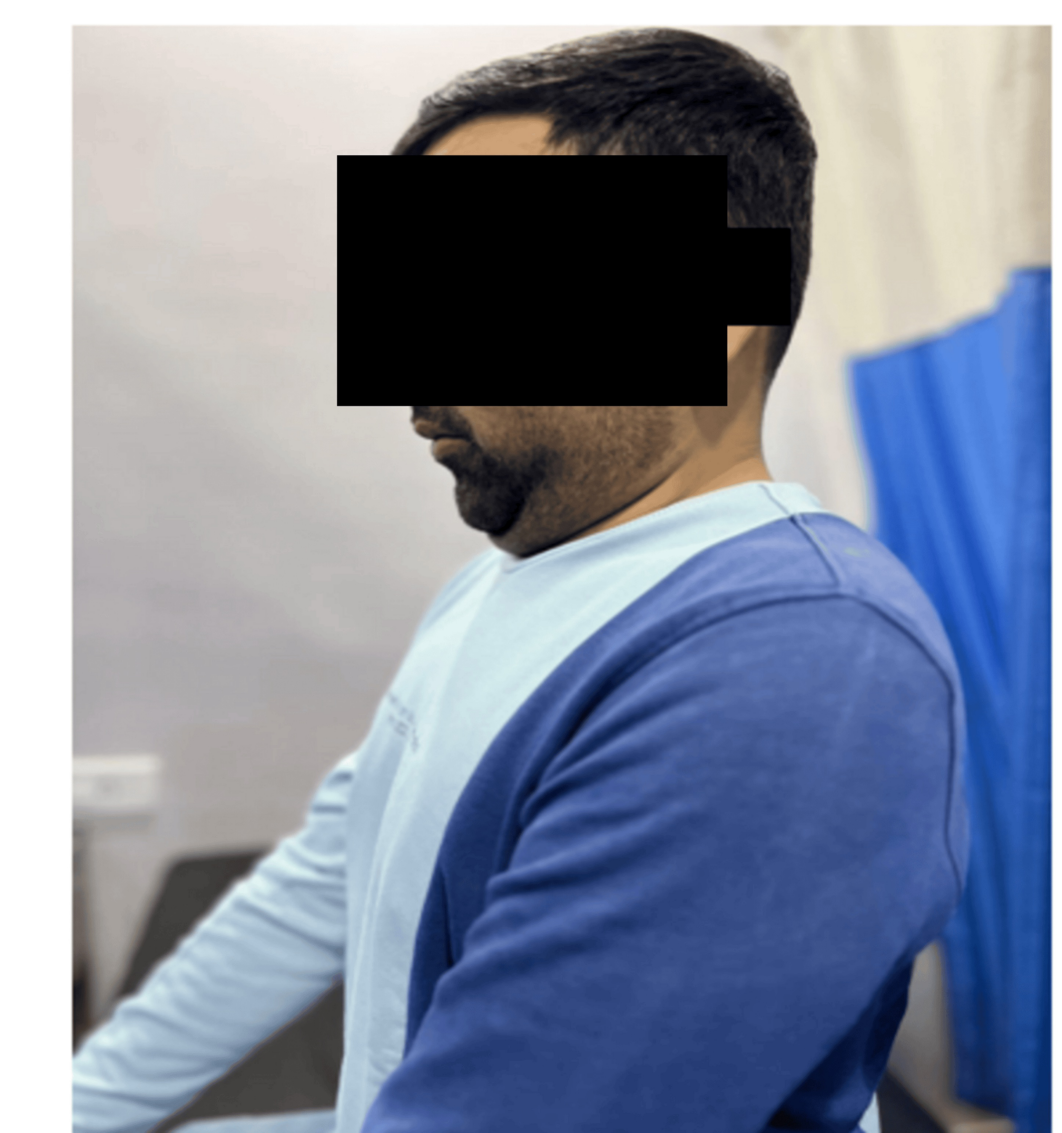
Demonstration of cervical retraction exercise in a seated position, used in the McKenzie approach-based intervention

Participants assigned to Group A performed a structured series of cervical exercises. These included cervical retraction held for 10 seconds and repeated 10 times (Figure 2). Neck extension in both supine and seated positions with 10 repetitions each, lateral flexion to the left and right sides for 10 repetitions per side, cervical rotation involving head turning for 10 repetitions, and cervical flexion in a seated position for 10 repetitions were also performed.

Group B: KCT 

Participants assigned to Group B underwent KCT exercises (Table 1) [22].

**Table 1:** Exercise program for Group B

Exercise	Repetitions	Sets	Hold duration
Thoracic flexion	3–5	2–3	10 seconds – 2 minutes
Horizontal retraction	3–5	2–3	10 seconds – 2 minutes
Arm extension	3–5	2–3	10 seconds – 2 minutes
Lift exercise	3–5	2–3	10 seconds – 2 minutes
Occiput lift (nodding)	3–5	2–3	10 seconds – 2 minutes
Forward head lean	3–5	2–3	10 seconds – 2 minutes
Cervical extension control	3–5	2–3	10 seconds – 2 minutes
Lower neck tilt	3–5	2–3	10 seconds – 2 minutes
Upper neck tilt	3–5	2–3	10 seconds – 2 minutes
Head turn	3–5	2–3	10 seconds – 2 minutes

**Figure 3:**
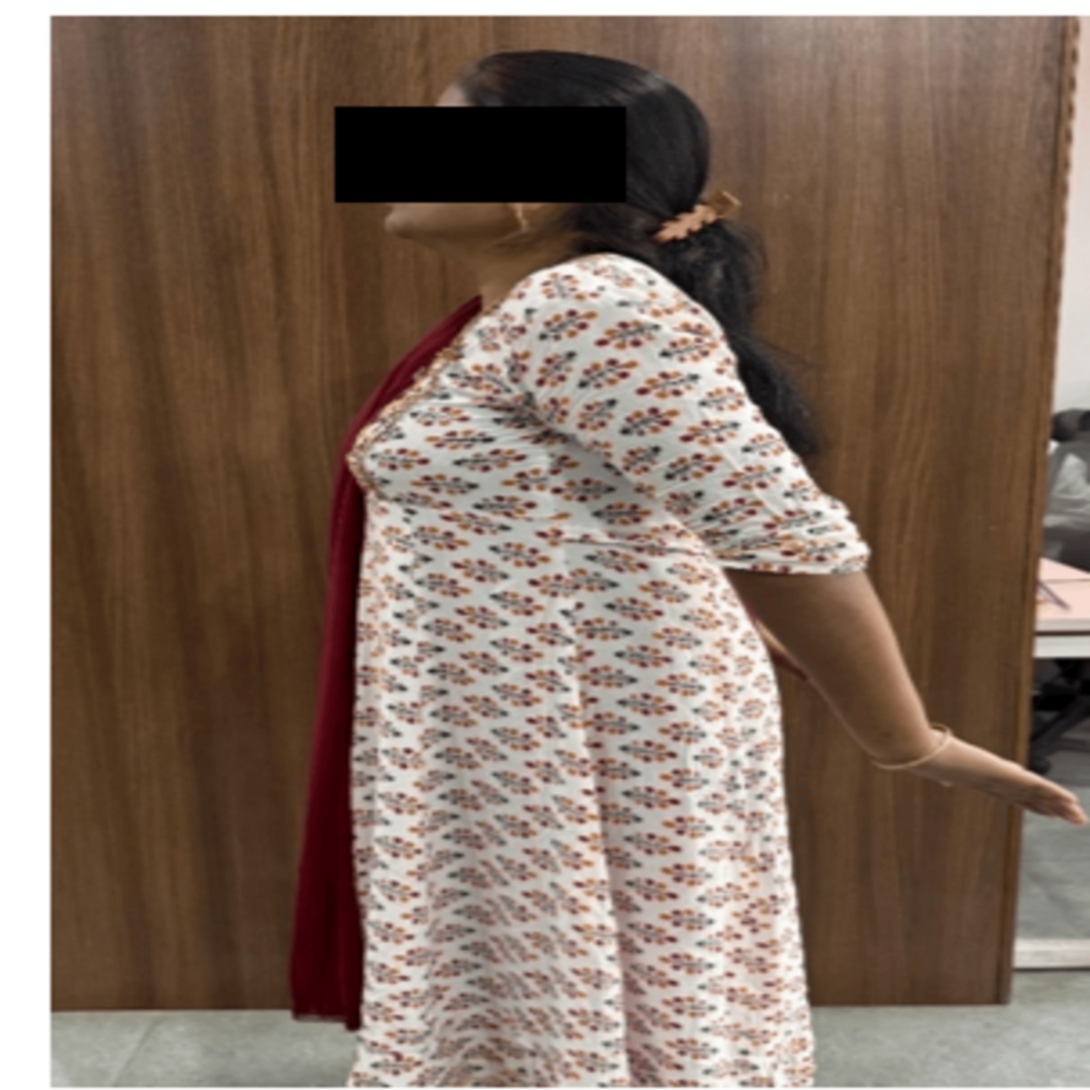
Arm extension exercise in the standing position used in kinetic control training exercise program

Figure 3 illustrates an arm extension exercise performed in a standing position as part of the KCT protocol. This exercise targets scapulothoracic stability and segmental control of the shoulder girdle, aiming to correct altered movement kinematics and reduce mechanical loading on cervical and thoracic structures during functional tasks.

Statistical analyses

Both manual analysis and software analysis using IBM SPSS Statistics software, version 26.0 were carried out. The numerical data were presented as means and standard deviations, and a paired t-test was employed to compare pre- and post-intervention data within the group. Continuous variables underwent normality testing. To assess significant changes over time, a repeated-measures analysis was conducted. A significance criterion of p < 0.0001 was set for all analyses to ensure robust statistical findings. This methodology provided a thorough evaluation of the data, integrating manual checks with advanced software analysis.

## Results

This study included 106 participants divided into two groups of 53 each (Group A and Group B) by simple random sampling. Group A received treatment based on McKenzie’s approach, and Group B received KCT four times weekly for six weeks. Among the 106 participants with CDS, the age of 30-50 years was found to be commonly affected. Sixty-one were female, and 45 were male (Table 2).

**Table 2:** Demographic characteristics of the participants in the study group

Variables	Number of Individuals
Age (years)	
30-40	31
41-50	22
Total	53
Gender	
Male	45
Female	61
Total	106

The pre- and post-test mean scores of VAS (at rest) within Group A and Group B showed that the pre-test and post-test mean scores for Group A were 2.702±1.42 and 0.574±0.49, respectively. For Group B, the corresponding mean scores were 2.74±1.40 and 0.21±0.41, respectively. The p-value for the pre-test and post-test scores was <0.0001, which was considered extremely significant. The pre- and post-test mean scores of VAS (on activity) within Group A and Group B showed that the pretest and the post-test mean for Group A were 6.468±1.62 and 5.57±1.90, whereas for Group B they were 5.74±2.25 and 0.44±0.50, respectively. Group A was considered very significant with a p-value of 0.0019, and group B was found to be extremely significant with a p-value < 0.0001 (Table 3).

**Table 3:** Comparison of pre- and post-test mean scores of VAS (at rest and during activity) within Groups A and B A paired t-test was used to calculate within-group analysis and p-values. The threshold for statistical significance is p <0.05. VAS: Visual Analog Scale

VAS (at rest)	Pre-test	Post test	Mean difference	P-value	T value
Group A	2.702±1.42	0.574±0.49	2.13	<0.0001	9.931
Group B	2.74±1.40	0.21±0.41	2.53	<0.0001	11.56
VAS (on activity)					
Group A	6.468±1.62	5.57±1.90	0.89	0.0019	3.30
Group B	5.74±2.25	0.44±0.50	5.3	<0.0001	16.10

Table 4 shows the pre-test and post-test values of the NDI score for both groups A and group B, respectively. Group A was considered significant with a p-value of 0.027, and group B was found to be extremely significant with a p-value <0.0001

**Table 4:** Comparison of pre- and post-test mean scores of NDI within Groups A and B A paired t-test was used to calculate within-group analysis and p-values. The threshold for statistical significance is p <0.05. NDI: Neck Disability Index

NDI	Pre-test	Post test	Mean difference	P-value	T value
Group A	24.95±5.64	23.89±6.99	1.06	0.0276	2.27
Group B	25±6.40	7.27±3.63	17.73	<0.0001	16.73

Table 5 shows the comparative analysis of cervical ROM using a paired t-test between Group A and Group B, indicating consistently superior outcomes in Group B. Both groups showed improvement in flexion and extension with p < 0.0001, though the changes in Group B were more pronounced and extremely significant. In left and right lateral flexion, Group A did not demonstrate significant changes (p = 0.0714 and p = 0.172, respectively), while Group B showed extremely significant improvements (p < 0.0001). Left rotation in Group A was marginally significant (p = 0.035), whereas Group B exhibited an extremely significant gain (p < 0.0001). Right rotation did not reach significance in Group A (p = 0.058) but was extremely significant in Group B (p < 0.0001). These findings confirm that Group B experienced extremely significant enhancements in cervical ROM across all movements.

**Table 5:** Comparison of pre- and post-test mean scores of cervical range of motion within Groups A and B A paired t-test was used to calculate within-group analysis and p-values. The threshold for statistical significance is p <0.05.

Movements	Group A (Mean ± SD)				Group B (Mean ± SD)			
	Pre-test	Post test	P-value	T value	Pre-test	Post test	P-value	T value
Flexion	52.04±1.73	53.91±2.11	<0.0001	2.27	52.51±1.66	59.53±0.50	<0.0001	28.31
Extension	67.23±2.14	69.23±2.77	<0.0001	4.07	66.42±2.70	74.06±0.79	<0.0001	18.90
Left lateral flexion	38±0.83	38.19±1.09	0.0714	1.84	39.72±1.83	45±0.83	<0.0001	17.20
Right lateral flexion	38±0.72	38.17±1.16	0.172	1.38	39±1.71	44.12±0.76	<0.0001	17.61
Left rotation	70.25±1.48	70.53±1.57	0.035	2.16	72.59±2.03	79.36±0.48	<0.0001	23.68
Right rotation	38.29±0.79	38.59±0.95	0.058	1.84	71.31±1.88	79.46±0.50	<0.0001	29.95

The post-test outcomes indicate significant differences using an unpaired t-test between Group A and Group B across all measured variables (Table 6). Cervical ROM showed greater improvements in Group B in all directions, with extremely significant p-values (<0.0001) for flexion, extension, lateral flexions, and rotations, confirming superior mobility gains in Group B. In terms of pain intensity, VAS scores at rest and during activity were substantially lower in Group B compared to Group A, both showing extremely significant differences (p < 0.0001), indicating greater pain relief in the experimental group. Furthermore, the NDI scores revealed a marked reduction in disability in Group B (7.27±3.63) compared to Group A (23.89±6.99), with an extremely significant p-value (<0.0001). Collectively, these findings demonstrate that the intervention provided to Group B was markedly more effective in improving cervical mobility, reducing pain, and enhancing functional outcomes compared to the treatment received by Group A.

**Table 6:** Comparison of post-test mean scores of the outcome measure between Groups A and B Unpaired t-test was used to calculate between-group analysis and p-values. The threshold for statistical significance is p <0.05. VAS: Visual Analog Scale; NDI: Neck Disability Index

Outcome measures	Group A (Mean ± SD)	Group B (Mean ± SD)	P-value
VAS (at rest)	0.574±0.49	0.21±0.41	<0.0001
VAS (on activity)	5.57±1.90	0.44±0.50	<0.0001
NDI	23.89±6.99	7.27±3.63	<0.0001
Flexion	53.91±2.11	59.53±0.50	<0.0001
Extension	69.23±2.77	74.06±0.79	<0.0001
Left lateral flexion	38.19±1.09	45±0.83	<0.0001
Right lateral flexion	38.17±1.16	44.12±0.76	<0.0001
Left rotation	70.53±1.57	79.36±0.48	<0.0001
Right rotation	38.29±0.95	79.46±0.50	<0.0001

## Discussion

The present study aimed to evaluate and compare the effects of KCT and the McKenzie approach in individuals diagnosed with CDS. Individuals with CDS typically present with mechanical neck pain, restricted cervical mobility, and impaired cervical movement mechanics due to underlying segmental dysfunctions. While the McKenzie approach has been widely used to centralize symptoms and improve mobility by utilizing repeated movements and patient education, it often focuses on directional preference and symptom management. KCT specifically targets altered movement mechanics by restoring segmental stability and improving movement efficiency during functional tasks. In this study, participants who received KCT showed a greater improvement in pain reduction, cervical ROM, and functional status compared to those who followed the McKenzie approach. While both interventions resulted in statistically significant improvements, the greater magnitude of benefit observed with KCT may be clinically meaningful for patients with CDS. These findings suggest that KCT not only addresses symptom relief but also provides a long-term strategy for movement correction and postural restoration, making it a more comprehensive intervention for managing CDS [22,23].

Comerford and Mottram (2001) introduced KC as a framework for assessing and addressing altered movement mechanics that contribute to musculoskeletal pain. Their model emphasizes the identification of subtle motor control impairments rather than simply focusing on joint range of motion or muscle strength. They proposed that faulty movement patterns could lead to biomechanical inefficiency, joint overload, and chronic symptoms, particularly in the cervical spine, where poor control of segmental motion results in instability and excessive strain on passive structures such as ligaments and discs. To counteract these issues, Comerford and Mottram advocated for low-load, high-repetition motor retraining aimed at restoring optimal joint kinematics. They stressed that exercises should be both direction-specific and task-specific, targeting the exact site and direction of the dysfunction. This approach aligns with the current study's findings, where KCT led to superior improvements in cervical function compared to the McKenzie approach [24].

Bhende et al. (2024), on text neck syndrome, investigated the effect of integrated postural training versus conventional exercise on pain, cervical ROM, posture, and proprioception in 80 individuals, using outcome measures such as VAS, NDI, cervical ROM, cervical joint position error (CPJE) testing, and the tragus-to-wall test. It found significant improvements in both groups, with superior proprioceptive gains in the postural training group. Similarly, our study evaluated the impact of KCT and the McKenzie approach on pain, function, and mobility in individuals with CDS. Both groups showed improvement, but KCT yielded significantly better outcomes in VAS, NDI, and cervical ROM [25].

Similarly, Fernández-de-las-Peñas et al. (2013) conducted a meta-analysis to evaluate the effectiveness of manual therapy and motor control exercises in the treatment of mechanical neck pain. They found that combining manual mobilizations with movement control retraining resulted in the most significant improvements in pain reduction, cervical ROM, and disability. In contrast, purely passive interventions, such as manual therapy alone, were found to be less effective. This highlights the importance of incorporating movement control retraining to restore cervical spine function, supporting the superiority of KCT, which inherently integrates active motor control correction and dynamic spinal stability [26].

Falla et al. (2017) further explored the outcomes of deep cervical flexor retraining using pressure biofeedback devices in their randomized controlled trial. They found that patients who received targeted deep muscle activation training exhibited significantly better results in pain reduction, muscular endurance, and disability scores compared to those who performed general strengthening exercises. These findings support the theoretical framework of KC, emphasizing the necessity of proper activation and endurance of specific stabilizing muscles for effective spinal control. This concept underpins the rationale for KCT’s effectiveness in addressing the motor control impairments commonly associated with CDS [27].

Clinical implications

The findings of this study underscore the clinical value of KCT in CDS. The superior outcomes observed in this study may be attributed to its focus on CDS, unlike prior studies on general neck pain. KCT targets long-term correction of abnormal movement kinematics, whereas the McKenzie approach primarily emphasizes symptom centralization. By addressing often-overlooked altered movement kinematics, KCT enhances cervical stability, functional mobility, and pain reduction through targeted correction of altered movement kinematics. Its patient-centered, progressive approach promotes adherence, self-management, and long-term outcomes. Given the rising incidence of neck pain, particularly among sedentary individuals, KCT serves as a vital intervention in both early and chronic stages of cervical dysfunction and complements manual therapy within a comprehensive rehabilitation framework.

Limitations and recommendations

The study has some limitations that include a single-center design, lack of assessor blinding, short-term follow-up, and reliance on self-reported adherence, which may affect the accuracy and generalizability of the findings. Future research should consider multicenter trials with extended follow-up periods, objective adherence monitoring, and exploration of combined interventions to enhance clinical outcomes in CDS.

## Conclusions

This study compared the effects of KCT and the McKenzie approach in managing cervical derangement. Both interventions were effective in reducing pain, improving cervical range of motion, and enhancing function. KCT provided greater reductions in pain and disability and larger gains in cervical mobility than the McKenzie approach in individuals with CDS. Larger, multicenter trials with longer follow-up periods are warranted to confirm these findings and strengthen the evidence base. These findings suggest that KCT may provide more sustainable improvements by targeting underlying altered movement kinematics, whereas McKenzie offers faster symptomatic relief.
